# Corrosive Sublimate as an Antiseptic

**Published:** 1884-03

**Authors:** 


### Corrosive Sublimate as an Antiseptic.

The corrosive chloride of mercury has long been known as an efficient antiseptic, and of late its use has been considerably extended in German hospitals, with results, it is claimed, superior to carbolic acid. A solution of the strength of one per cent. is employed in the gynæcological clinic at Breslau for washing the hands before examinations, etc., cleansing instruments, disinfecting basins, etc. Dr. Kustner, of Jena, uses a solution of one part to 5,000 for medical inspections in the cystitis of lying-in women, with excellent results.

